# Proteomic Profiling of Archived Tissue of Primary Melanoma Identifies Proteins Associated with Metastasis

**DOI:** 10.3390/ijms21218160

**Published:** 2020-10-31

**Authors:** Andrew Shapanis, Chester Lai, Mathew Sommerlad, Erika Parkinson, Eugene Healy, Paul Skipp

**Affiliations:** 1 Centre for Proteomic Research, Biological Sciences, University of Southampton, Southampton SO17 1BJ, UK; andy.shapanis@soton.ac.uk (A.S.); E.P.Parkinson@soton.ac.uk (E.P.); 2 Dermatopharmacology, Clinical and Experimental Sciences, Faculty of Medicine, University of Southampton, Southampton SO16 6YD, UK; C.Y.Lai@soton.ac.uk (C.L.); E.Healy@soton.ac.uk (E.H.); 3 Dermatology, University Hospital Southampton NHS Foundation Trust, Southampton SO16 6YD, UK; 4 Histopathology, University Hospital Southampton NHS Foundation Trust, Southampton SO16 6YD, UK; mathew.sommerlad@uhs.nhs.uk

**Keywords:** proteomics, melanoma, metastasis, FFPE, formalin fixed paraffin embedded

## Abstract

Formalin-fixed paraffin embedded (FFPE) clinical tissues represent an abundant and unique resource for translational proteomic studies. In the US, melanoma is the 5th and 6th most common cancer in men and women, respectively, affecting over 230,000 people annually and metastasising in 5–15% of cases. Median survival time for distant metastatic melanoma is 6–9 months with a 5-year-survival of < 15%. In this study, 24 primary FFPE tumours which have metastasised (P-M) and 24 primary FFPE tumours which did not metastasise (P-NM) were subjected to proteomic profiling. In total, 2750 proteins were identified, of which 16 were significantly differentially expressed. Analysis of TCGA data demonstrated that expression of the genes encoding for 6 of these 16 proteins had a significant effect on survival in cutaneous melanoma. Pathway analysis of the proteomics data revealed mechanisms likely involved in the process of melanoma metastasis, including cytoskeleton rearrangement, extracellular changes and immune system alterations. A machine learning prediction model scoring an AUC of 0.922, based on these 16 differentially expressed proteins was able to accurately classify samples into P-M and P-NM. This study has identified potential biomarkers and key processes relating to melanoma metastasis using archived clinical samples, providing a basis for future studies in larger cohorts.

## 1. Introduction

Clinical tissue samples can be preserved in formaldehyde, embedded in paraffin wax, and stored at room temperature indefinitely without significant degradation occurring. This has resulted in huge biobanks of archived samples, frequently along with retrospective clinical information of a disease for which the clinical outcome and therapy responses are known, thus representing a valuable resource for biomarker discovery and the molecular profiling of disease pathways [1,2]. The practical utility of using formalin-fixed paraffin embedded (FFPE) samples for proteomic studies has previously been hampered by the adverse effects of formaldehyde fixation, making the efficient extraction of proteins a challenge. During the FFPE process, samples are incubated in formaldehyde for many hours resulting in the ubiquitous formation of cross linkages, most notably between primary amines. Unlike fresh/frozen samples, which are relatively straight forward to process, FFPE samples require additional steps to reduce these cross-linkages and enable downstream analysis. However, recent advances in Mass spectrometry (MS)-based proteomic methods now allow in-depth proteomic analysis of FFPE tissues [3]. The availability of this unique resource has motivated us to perform a proteomic analysis of primary melanoma in relation to the development of subsequent metastases using FFPE archived primary melanoma tumour samples in combination with their associated clinical outcome data.

Globally, cutaneous melanoma is thought to account for 1.7% (232,100) of all newly diagnosed cases of primary malignant cancers and 0.7% of all cancer deaths each year [4]. The incidence of melanoma differs widely amongst countries, with New Zealand and Australia having the highest rate at 35.8 per 100,000 person-years and 34.9 per 100,000 person-years, respectively [5]. The mortality rate of those in high risk countries like New Zealand and Australia is also elevated, with observed rates at 4.7 per 100,000 person-years and 4.0 per 100,000 person-years, respectively [5].

Melanoma metastasis is considered to occur in 5–15% of all melanoma cases [6,7,8], with one study of > 3000 patients reporting that 15.5% of all melanomas progressed to metastatic melanoma and that 57.3% of all patients who developed distant metastasis died at the time of reporting [8]. The risk of mortality is directly associated with metastasis since 5-year survival rates for distant metastatic melanoma are as low as 15%, with a median survival of 6–9 months [9,10].

Much of the current literature regarding melanoma metastasis focuses on comparisons of primary tumours and their metastases. Although a few studies have investigated FFPE melanoma samples using proteomics [11,12,13], to our knowledge, there have been no investigations comparing primary melanomas that have metastasised with primary melanomas that have not metastasised, a comparison which could help to inform on proteins and mechanisms involved in the progression to metastatic melanoma.

In this study, we have used global proteomic profiling of primary melanoma tumours, which had either metastasised or not metastasised, to identify potential biomarkers and possible mechanisms relating to the development of melanoma metastasis. These results highlight several key molecular processes which are likely involved in melanoma metastasis with a panel of proteins identified that could potentially be used to predict metastasis in unknown tumours.

## 2. Results

**Figure 1:**
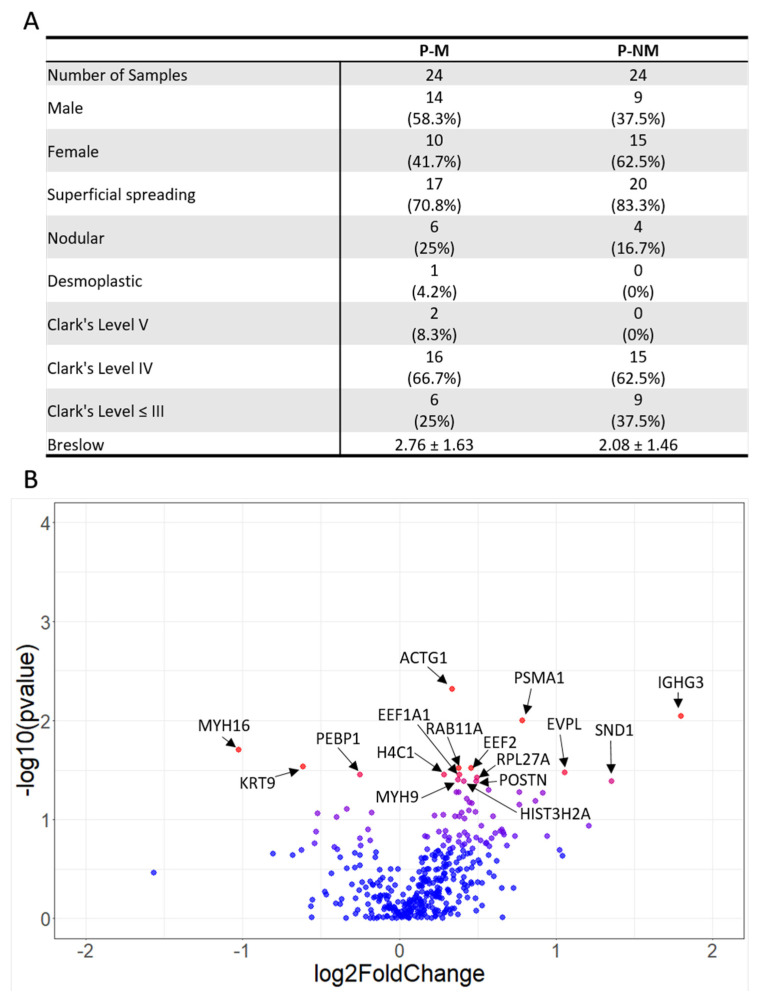
Proteomic analysis of primary melanomas which had metastasised (P-M) and those which had not metastasised (P-NM) reveals 16 significantly differentially expressed proteins. (**A**) clinical characteristics of P-M and P-NM samples. (**B**) Volcano plot of proteomic data. Pvalues were obtained by Mann–Whitney U test and fold changes by dividing the mean of each P-M protein by the mean of the P-NM protein. Blue = *p* > 0.05, red = *p* < 0.05.

We compared the proteomic profiles of primary melanomas which had metastasised (P-M) with primary melanomas that had not metastasised (P-NM). A total of 48 samples were used, 24 in each group (P-M and P-NM) (Figure 1A). There were marginally more males in the P-M group compared to the P-NM group. The majority of melanomas in both P-M and P-NM groups were superficial spreading melanoma and Clark’s level IV. Samples were stratified for Breslow thickness and showed no significant difference between the two groups.

**Figure 2:**
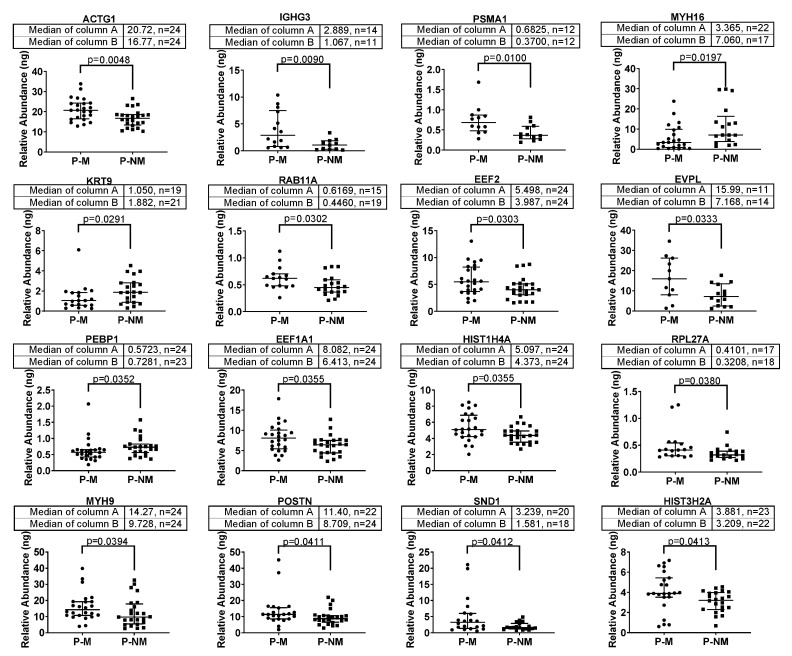
Dot plots showing individual tumour level of expression for each of the 16 significantly differentially expressed proteins in P-M and P-NM melanomas. Expression data for each protein were plotted using Prism, V8 (Graphpad, San Diego, CA, USA).

In total, 2750 proteins were identified from the 48 FFPE samples, with 16 of these proteins (listed in the volcano plot, Figure 1B) identified as significantly differentially expressed between P-M and P-NM. Dot plots for the individual tumour expression of each of these 16 significantly differentially expressed proteins are shown in Figure 2.

**Figure 3:**
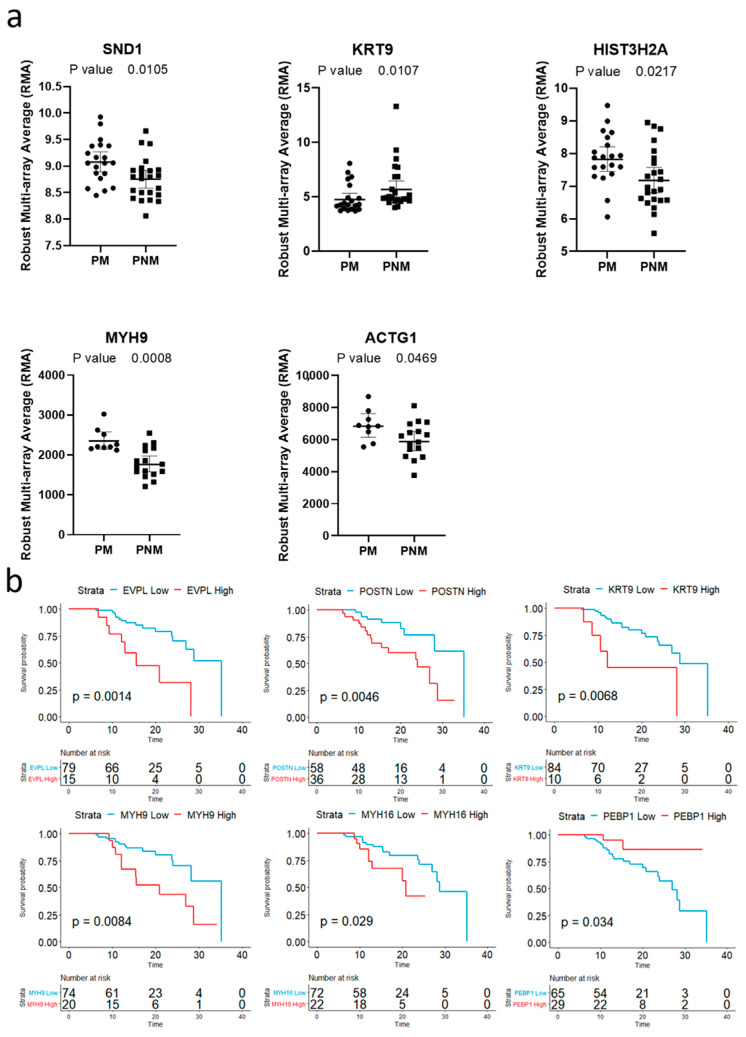
Validation of differentially expressed proteins using two NCBI GEO datasets and TCGA survival analysis. (**a**) Primary melanoma samples which could be inferred as either P-M or P-NM from GEO datasets; GSE15605 and GSE46517, were analysed separately for the 16 differentially expressed proteins identified via mass spectrometry. Median with interquartile range shown as error bars. (**b**) Survival analysis of cutaneous melanoma data from TCGA indicated that genes encoding for 6 proteins that were significantly differentially between P-M and P-NM have a significant effect on 3-year survival in patients with cutaneous melanoma.

Two datasets from the National Center for Biotechnology Information’s Gene Expression Omnibus (NCBI GEO, datasets; GSE15605 and GSE46517) were used to validate these significantly differentially expressed proteins. Genes; Staphylococcal nuclease domain-containing protein 1 (*SND1*), Keratin 9 (*KRT9*), Histone H2A type 3 (*HIST3H2A*), Myosin-9 (*MYH9*) and Actin Gamma 1 (*ACTG1*) were significantly differentially expressed between PM and PNM in these data sets. Furthermore, to determine whether these significantly differentially expressed proteins that were associated with the development of metastases from primary melanoma were associated with a poorer clinical outcome, survival analysis of the cutaneous melanoma cohort from The Cancer Genome Atlas (TCGA) was performed. This analysis identified that expression of the genes encoding for 6 of the 16 significantly differentially expressed proteins were associated with reduced survival over a 3-year period (Figure 3); these comprised Envoplakin (*EVPL*), Periostin (*POSTN*), *KRT9*, *MYH9*, myosin heavy chain 16 (*MYH16*) and Phosphatidylethanolamine-binding protein 1 (*PEBP1*).

**Figure 4:**
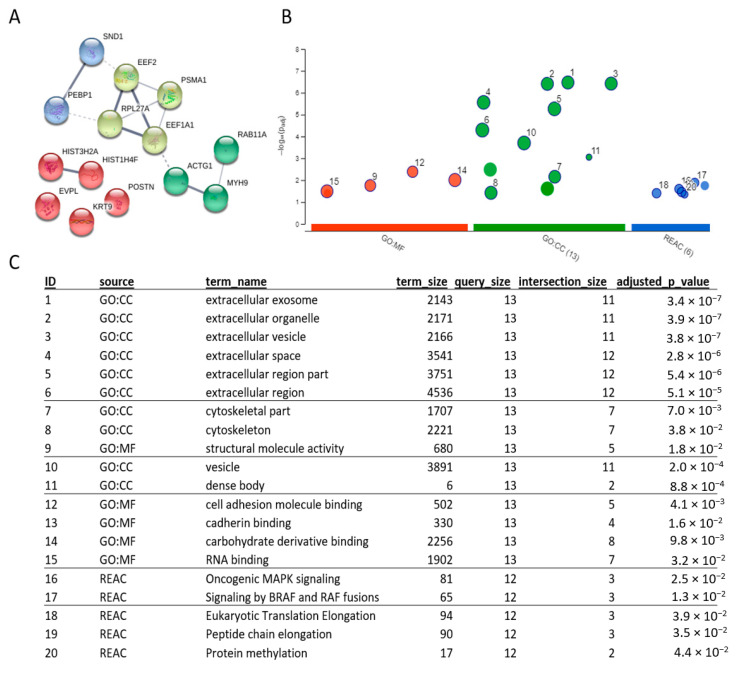
Analysis of significantly differentially expressed proteins highlighted processes involved in metastasis. (**A**) String analysis of significantly differentially expressed proteins. Cluster analysis based on protein-protein interactions revealed 4 clusters. (**B**,**C**) Gene ontology enrichment analysis of significantly differentially expressed proteins highlighted several areas of ontology including extracellular space, cytoskeletal, vesicles and binding. GO; Gene ontology. CC; cell compartment. MF; molecular function. REAC; reactome.

Protein network analysis of the significantly differentially expressed proteins was performed using STRING, v11 (Figure 4A). Using the STRING clustering function, four distinct clusters were identified, with the main clusters consisting of functions associated with translational and structural components. Functional and enrichment analyses of the significantly differentially expressed proteins were performed using G:Profiler. These revealed that these proteins were enriched in biological processes and molecular functions associated with the Mitogen-activated protein kinase (*MAPK*) pathway, translation/protein synthesis, and a range of binding functions (including intracellular and cell-to-cell) (Figure 4B,C). The most enriched ontologies in the Gene Ontology cellular compartment analysis were extracellular space, cytoskeletal and vesicles.

**Figure 5:**
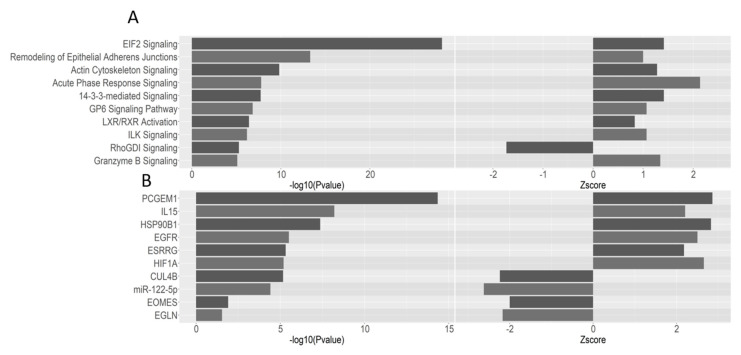
Ingenuity pathway analysis (IPA) of P-M vs. P-NM proteomics data. Unique protein IDs present in at least 50% of samples were analysed using IPA with fold changes and *p* values used as metrics. (**A**) IPA pathway analysis. (**B**) IPA upstream regulator analysis.

Ingenuity pathway analysis (IPA) of all unique proteins identified within at least 50% of samples, along with their p values and fold changes, reflected the gene ontology enrichment analysis (Figure 5), highlighting translation/protein synthesis (EIF2 signalling), cytoskeletal remodelling (epithelial adherens junctions, actin cytoskeleton signalling) and extracellular signalling (Integrin-linked kinase, ILK) as enriched processes. Activation of the signalling pathways associated with acute phase response, granzyme B, IL15, the upstream modulator, *HSP90B1* and inhibition of the upstream modulator EOMES also indicated immune related mechanisms in development of melanoma metastasis. Other interesting areas of enrichment included activation and inhibition of upstream hypoxia associated proteins including Hypoxia-inducible factor 1-alpha (*HIF1a*) and egl-9 family hypoxia inducible factor 1 (*EGLN*), respectively.

**Figure 6:**
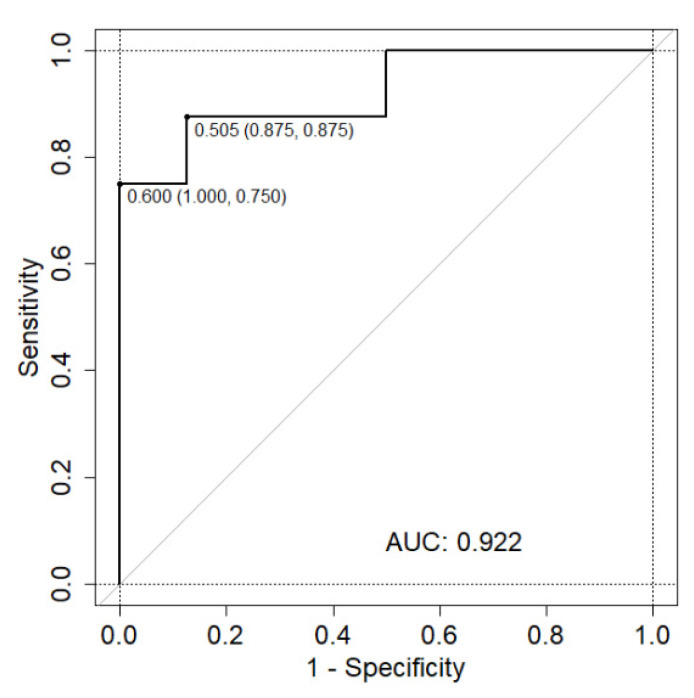
Machine learning on the proteomics data generated a model capable of predicting melanoma metastasis. Significantly differentially expressed proteins were trained on 67% of the data (32 melanoma samples) using 5-fold cross validation repeated 3 times. Missing values were median imputed. Several different algorithms were tested for performance by ROC analysis and then the best performing tested on the 33% of data held out for testing (16 melanoma samples).

A Distance Weighted Discrimination with a Radial Basis Function Kernel machine learning model was used to create a predictive model based upon the 16 significantly differentially expressed proteins to classify samples as either P-M or P-NM. Figure 6 shows the area under the Receiver Operating Characteristic (ROC) curve, correctly classifying P-M samples with excellent performance (AUC = 0.922). There were two optimal thresholds (that is the threshold that achieved the highest sum sensitivity/specificity). The first achieved a sensitivity of 75% and specificity of 100%, the second achieved a sensitivity and specificity of 88%. This model highlights the potential predictive power of these proteins, but warrants further investigation in a larger independent cohort. 

## 3. Discussion

The majority of human primary tumours are excised where possible in order to minimise the development of subsequent metastases. The excised tissue is processed to produce FFPE samples, thus preserving the tissue and the tissue architecture to allow a diagnosis to be made, along with any additional immunohistochemistry investigations to support the diagnosis and possibly indicate prognoses. As this procedure is commonplace across many countries, the resulting samples represent an extremely large worldwide biobank of FFPE tissue pathology from a multitude of cancers and other non-neoplastic diseases [1]. Furthermore, complementary clinical information is often available, thus providing a valuable resource for studies looking for biomarkers, possible therapeutic targets, etc.

In this study, we utilised FFPE melanoma samples along with clinical information to perform a retrospective study on primary melanomas to identify proteins and pathways associated with the progression of melanoma to metastatic melanoma. Using a quantitative proteomic approach, we compared 24 FFPE primary melanomas that had metastasised (P-M) and 24 FFPE primary melanomas which had not metastasised (P-NM). Breslow depth is a well-known prognostic marker for cutaneous melanoma [14,15], and, therefore, samples were stratified to ensure there was no significant difference in Breslow thickness between P-M and P-NM groups.

We identified 2750 proteins across the FFPE primary tumours and statistical analysis revealed 16 significantly differentially expressed proteins between P-M and P-NM (Figure 2). Several of these proteins were unable to be quantified across all 24 P-Ms and 24 P-NMs. This may be attributed to the complex nature of FFPE samples and the extensive number of cross-linkages formed during the fixation process. Cross-linked peptides will often result in a peptide not being matched to a protein because of unknown peptide modifications which are unaccounted for during the database search. Furthermore, these cross-linkages increase the complexity of the sample, which subsequently increases the signal: noise threshold [16]. Although many imputation methods exist, we considered the most conservative approach was to use the available data without imputation as it is often more robust and is less likely to suffer from any other biases [17]. Many of these 16 proteins have previously been shown to influence metastasis in other cancers, for instance, ACTG1 in liver cancer [18], Immunoglobulin heavy constant gamma 3 (*IGHG3*) in breast cancer [19], Proteasome subunit alpha type-1 (*PSMA1*) in colon cancer [20], Ras-related protein Rab-11A (*RAB11a*) in pancreatic cancer [21], PEBP1 and Eukaryotic translation elongation factor 1 alpha 1 (*EEF1A1*) in hepatocellular carcinoma [22,23], MYH9 in colorectal [24] and ovarian cancer [25], POSTN in melanoma [26], hepatocellular carcinoma [27] and breast cancer [28] and SND1 in breast cancer [29] and cervical cancer [30]. Furthermore, five of these proteins were identified as significantly differentially expressed between PM and PNMs in two NCBI GEO datasets and the gene expression of 6 of these 16 significantly differentially expressed proteins (i.e., EVPL, POSTN, KRT9, MYH9, MYH16 and PEBP1) each had a significant effect on 3-year survival rates in cutaneous melanoma as identified from the TCGA database. Interestingly, here we report that both protein and mRNA expression (via microarray) of KRT9 is significantly increased in PNMs compared to PMs but, when investigating mRNA (via RNAseq) in terms of survival, an increase in KRT9 is associated with poorer outcome.

STRING, GO and IPA of the proteomics data highlighted several key processes likely to be involved in melanoma metastasis. Translation and protein synthesis were identified by both gene ontology enrichment (Figure 4C) and pathway analysis (Figure 5A) and could be accredited to the loss of differentiation (and therefore a change in which proteins are produced and at what amounts) indicative of metastatic tumours [31,32]. Cell migration is also a key step in tumour progression and metastasis [33], occurring via a complex mechanism whereby the migrating cell remodels the cytoskeleton (Figure 4) to produce invadopodium which then break down the extracellular matrix to allow invasion into the surrounding tissue. A dysregulation of Rho signalling (Figure 5) is known to influence cell polarity and thus, in this instance, could be promoting progression by increasing invasiveness [34]. Furthermore, an activation of Epidermal growth factor receptor (*EGFR*) predicted by IPA and Epidermal growth factor (*EGF*) has been shown to promote invadopodium formation, aiding invasion and migration [35]. Additionally, an enrichment in intracellular vesicles, dense bodies and extracellular exosomes/organelles and vesicles were identified in GO (Figure 4C), suggesting possible extracellular trafficking. Related to this, it is known that tumour-derived exosomes can promote the development of metastasis from cancer, including from melanoma, in distant organs [36,37].

It is recognised that the immune system plays an important role in tumorigenesis and cancer metastases, including in skin cancer [38,39]. Indeed, it has been shown that immunosuppressed individuals have an increased risk of developing melanoma [40], and that immunosuppressed individuals are at much greater risk of developing metastasis than immunocompetent individuals [41]. In the current study, IPA revealed upregulation of IL15 signalling, an interleukin known to promote the activation and proliferation of natural killer cells and T cells [42,43]. Furthermore, the protein HSP90B1, which has been reported as playing a critical role in the regulation of natural killer and T cells [44] was predicted by IPA as an activated upstream regulator. In addition, an increase in granzyme B signalling (an important protease in the CD8+ cytotoxic T cells arsenal) was noted; however, IPA also indicated inhibition of signalling of eomesodermin (*EOMES*) which is also highly expressed in CD8+ T cells but seems to play a complex role in antitumour responses with some evidence for the promotion of adaptive immune responses against cancer but also in promoting CD8+ T cell exhaustion [45,46]. Furthermore, the aforementioned enrichment in extracellular trafficking may also involve the immune response, because recent evidence suggests that melanoma-derived exosomes can suppress effector cells in patients with this cancer [47].

Many studies in the published literature report on differences between benign/precancerous and primary and/or metastastic tumours; however, we took a different approach where we conducted proteomics on primary tumours which had metastasised and primary tumours that had not metastasised. As our study was performed on primary tumours, it presented the opportunity to investigate whether the significantly differentially expressed proteins could reliably be used to classify samples into either P-M or P-NM (i.e., metastasising or non-metastasising primary tumours). Using a Distance Weighted Discrimination with a Radial Basis Function Kernel machine learning model, an AUC of 0.922 was achieved suggesting these proteins as potential predictive protein biomarkers. Admittedly, these potential biomarkers will need to be investigated in a larger cohort to further assess their predictive power and utility. Nonetheless, access to the large valuable resource of FFPE tumours in healthcare opens up the opportunity for validating and identifying further prognostic biomarkers of melanoma.

This study has identified a number of proteins and pathways associated with the progression of melanoma to metastatic melanoma and has highlighted several potential biomarkers that could be further developed to predict the metastatic potential of primary melanomas from routinely collected FFPE samples.

## 4. Materials and Methods

### 4.1. Tissue Samples

FFPE human primary melanomas were acquired from Histopathology, University Hospital Southampton NHS Foundation Trust (UHS-NHSFT) under local research ethics committee approval (South Central Hampshire B National Research Ethics Service Committee; LREC number 07/H0504/187, amendment approved 7th July 2014). Samples were categorized as primary melanomas that metastasised (P-M) or primary melanomas that had not metastasised (P-NM), with the latter based on an absence of metastasis as determined by Dermatology UHS-NHSFT at and/or beyond 5-years post-surgical excision of the primary tumour. Samples were stratified for Breslow thickness so that there was no significant difference in this parameter between the two groups.

### 4.2. Microdissection and Processing of FFPE Tumour Samples

Three 10 µm FFPE sections per sample were cut and mounted onto glass slides before being deparaffinised, rehydrated, and stained with Mayer’s hematoxylin. The melanoma and associated immune infiltrate were microdissected and placed into 100 µL protein extraction buffer (containing 50 mM ammonium bicarbonate, 5 mM dithiothreitol, and 0.2% RapiGest SF (Waters, UK)). Following heating at 105 °C for 30 min, samples were cooled on ice for 5 min, re-heated to 80 °C for 2 h, placed on ice for 5 min again before reduction using 5 mM dithioerythritol at 60 °C for 30 min. To alkylate the samples, a final concentration of 15 mM iodoacetamide was added to each sample and incubated for 30 min, in the dark, at room temperature. Samples were subsequently digested overnight at 37 °C with 1µg trypsin. To cleave the Rapigest, a final concentration of 0.5% TFA was added to each sample and incubated at 37 °C for 30 min. Samples were next centrifuged at 15,000× *g* for 15 min. Supernatants were collected and lyophilised using an Eppendorf Concentrator-5301, then reconstituted in 150 µL of buffer A (0.5% formic acid in LC/MS grade water). C18 reverse phase clean-up was performed on the digested peptides using an EmporeTM C18 plate (Sigma, Poole, UK), where samples were bound to the membrane and washed twice with buffer A, before being eluted with 80% acetonitrile/water. Samples were subsequently lyophilised and reconstituted in buffer A ready for mass spectrometry analysis.

### 4.3. Liquid Chromatography Mass Spectrometry (LC-MS^E^)

Samples were analysed using a nanoACQUITY UPLC system (Waters, Manchester, UK) coupled to a Synapt G2-Si high-resolution mass spectrometer (Waters, UK) operating in MSE mode with ion mobility enabled. Peptide extracts were trapped onto a Symmetry-C18 180 µm x 20 mm trap column (Waters, UK) using buffer A and subsequently separated on a 75 µm I.D x 250 mm, 1.7 µm particle size, C18 analytical column (Waters, UK) over a 150-min linear gradient of 1 to 65% of buffer B (0.1% formic acid in acetonitrile (*v/v*)). A constant flow rate of 300 nl/min was used and 20 µL/min for trapping. Samples were introduced into the mass spectrometer using electrospray ionisation. Three, one-hour blank gradients between each sample were performed to eliminate carryover between samples. Samples were randomly batched into groups of 12. Standards were analysed at the beginning and end of every batch to assess instrument performance. Raw mass spectrometry data were processed using ProteinLynx Global Server 3.0 (Waters, UK) and searched against the human SwissProt database (November 2016 - 20,214 entries) allowing for deamidation of asparagine and glutamine, oxidation of methionine, and hydroxymethylation of cysteine with fixed modifications of carbamidomethylation of cysteine. The false discovery rate (FDR) was estimated with randomized decoy database searches and was filtered to 1% FDR at the protein level.

### 4.4. Data and Statistical Analysis

Only proteins detected in ≥50% of samples were subsequently analysed. Protein concentrations were normalised to median protein concentration for each sample. Volcano plots were created using R, Version 3.5.0. *p* values were calculated using Mann–Whitney U test for significance. Inferno RDN V1.1.7 was used to perform statistical analysis on proteomics data. R packages caret, pROC and doParallel were utilised for machine learning. The open source R package, gprofiler2 ver0.1.8 was used for gene ontology analysis and pathway analysis was performed using Ingenuity pathway analysis (IPA) with the results exported to create graphs in R.

### 4.5. NCBI GEO and TCGA Analysis

NCBI GEO datasets GSE15605 and GSE46517 were downloaded using the GEOquery (ver 2.58.0) package in R. Primary melanomas were extracted from the expression data and sorted into PM and PNM using the metadata provided. Patients in GSE46517 that were identified as having other cancers or those which died from unrelated reasons, were removed. Repeat gene probes were mean averaged. T test and Wilcoxon signed rank tests were performed depending on the data’s gaussian distribution. Plots were made in GraphPad prism. Using the FirebrowseR package for R (ver 1.1.35) and the Firebrowse webpage, TCGA clinical and gene expression data were obtained for the 16 significantly differentially expressed proteins identified between P-M and P-NM. The RSEM scaled estimate gene expression output was multiplied by 1^106 to calculate the transcripts per million, which was then used to plot survival curves. Survival data of 3 years were plotted in R using the Survminer and Survival packages. The optimal cut point for each group was calculated using the Survminer package which utilizes the maximally selected rank statistics from the maxstat R package.

### 4.6. Machine Learning

Proteomic data were split into training and test datasets, consisting of 67% [32] and 33% [16], respectively. Machine learning was performed on the training set using 5-fold cross validation repeated 3 times. For the 16 significantly differentially expressed proteins, a Distance Weighted Discrimination with a Radial Basis Function Kernel model was employed. Each resample was tuned using automatic tuning with a maximum tune length of 50.

## Abbreviations

AUC	Area under the curve
FFPE	Formalin fixed, paraffin embedded
FDR	False discovery rate
GO	Gene ontology
IPA	Ingenuity pathway analysis
LC-MS	Liquid chromatography – mass spectrometry
P-M	Primary metastatic
P-NM	Primary non-metastatic
ROC	Receiver operating characteristic curve
TCGA	The Cancer Genome Atlas
